# Critical respiratory failure in pregnancy complicated with COVID-19: A case report

**DOI:** 10.1016/j.crwh.2021.e00309

**Published:** 2021-03-23

**Authors:** Yukiko Chinen, Yoshino Kinjyo, Keiko Mekaru, Tadatsugu Kinjo, Yuuri Higure, Takeshi Kinjo, Kazuya Miyagi, Hisako Yamada, Hitoshi Masamoto, Hideki Goya, Tomohide Yoshida, Sakiko Maeshiro, Masashi Nakamatsu, Jiro Fujita, Yoichi Aoki

**Affiliations:** a Department of Obstetrics and Gynecology, Graduate School of Medicine, University of the Ryukyus, 207 Uehara Nishihara, Okinawa 903-0215, Japan; b Department of Infectious, Respiratory, and Digestive Medicine, University of the Ryukyus, 207 Uehara Nishihara, Okinawa 903-0215, Japan; c Department of Pediatrics, Graduate School of Medicine, University of the Ryukyus, 207 Uehara Nishihara, Okinawa 903-0215, Japan; d Infection Control Team, University of the Ryukyus Hospital, 207 Uehara Nishihara, Okinawa 903-0215, Japan

**Keywords:** COVID-19, Pneumonia, Pregnancy, Respiratory failure

## Abstract

The case is presented of a 29-year-old primiparous woman who was COVID-19-positive at 34 weeks of gestation and who developed severe acute respiratory distress syndrome. After a four-day history of fever and mild dyspnea, she was referred to hospital. Ciclesonide, dexamethasone, heparin sodium, and sulbactam/ampicillin were initiated, followed by remdesivir and tocilizumab. On the fourth day after admission (at 34 weeks 5 days of gestation), respiratory failure required ventilator management. An emergency cesarean section was performed and a 2565-g male infant was delivered with an Apgar score of 8/8 and negative COVID-19 status. However, on the following day the patient's respiratory condition deteriorated and mechanical ventilation was initiated. Subsequently, her respiratory condition quickly improved and mechanical ventilation was terminated 4 days after intubation. She was discharged 12 days after cesarean delivery. Our case provides additional evidence that raises concerns regarding the unfavorable maternal consequences of COVID-19 infection during pregnancy.

## Highlights

• A COVID-19-positive woman at 34 weeks of gestation presented with respiratory failure.
• An emergency cesarean section was performed at 34 weeks and 5 days of gestation.
• The patient's respiratory condition deteriorated the following day.
• Mechanical ventilation was initiated and was terminated four days after intubation.
• The case illustrates the unfavorable maternal consequences of COVID-19 infection during pregnancy.

## 1 Introduction

Coronavirus disease-2019 (COVID-19) can cause rapid progression of viral pneumonia, leading to acute respiratory distress syndrome (ARDS) requiring intubation. In the early period of the COVID-19 pandemic, the limited data that were available on COVID-19 infection in pregnancy indicated largely mild disease and good treatment outcomes with infections in the third trimester [1]. However, recent reports have demonstrated that COVID-19 infection during pregnancy is associated with increased risks of hospitalization, admission to an intensive care unit (ICU), and management with mechanical ventilation, but not with death [2]. Nevertheless, maternal deaths due to COVID-19 have been reported in the second or third trimesters [3]. Moreover, pregnant women may be at an increased risk of illness from COVID-19 compared with nonpregnant women. Preexisting comorbidities, high maternal age, and high body mass index seem to be risk factors for severe COVID-19. Rates of preterm birth are higher in pregnant women with COVID-19 than in pregnant women without the disease [4]. Pregnant women with COVID-19 who experience respiratory failure present multiple management challenges.

Here, the case of a COVID-19-positive, 29-year-old primiparous Japanese woman at 34 weeks of gestation presenting with severe respiratory compromise is presented. She experienced critical respiratory failure. Her clinical course and medical management are described.

## 2 Case Presentation

**Fig. 1:**
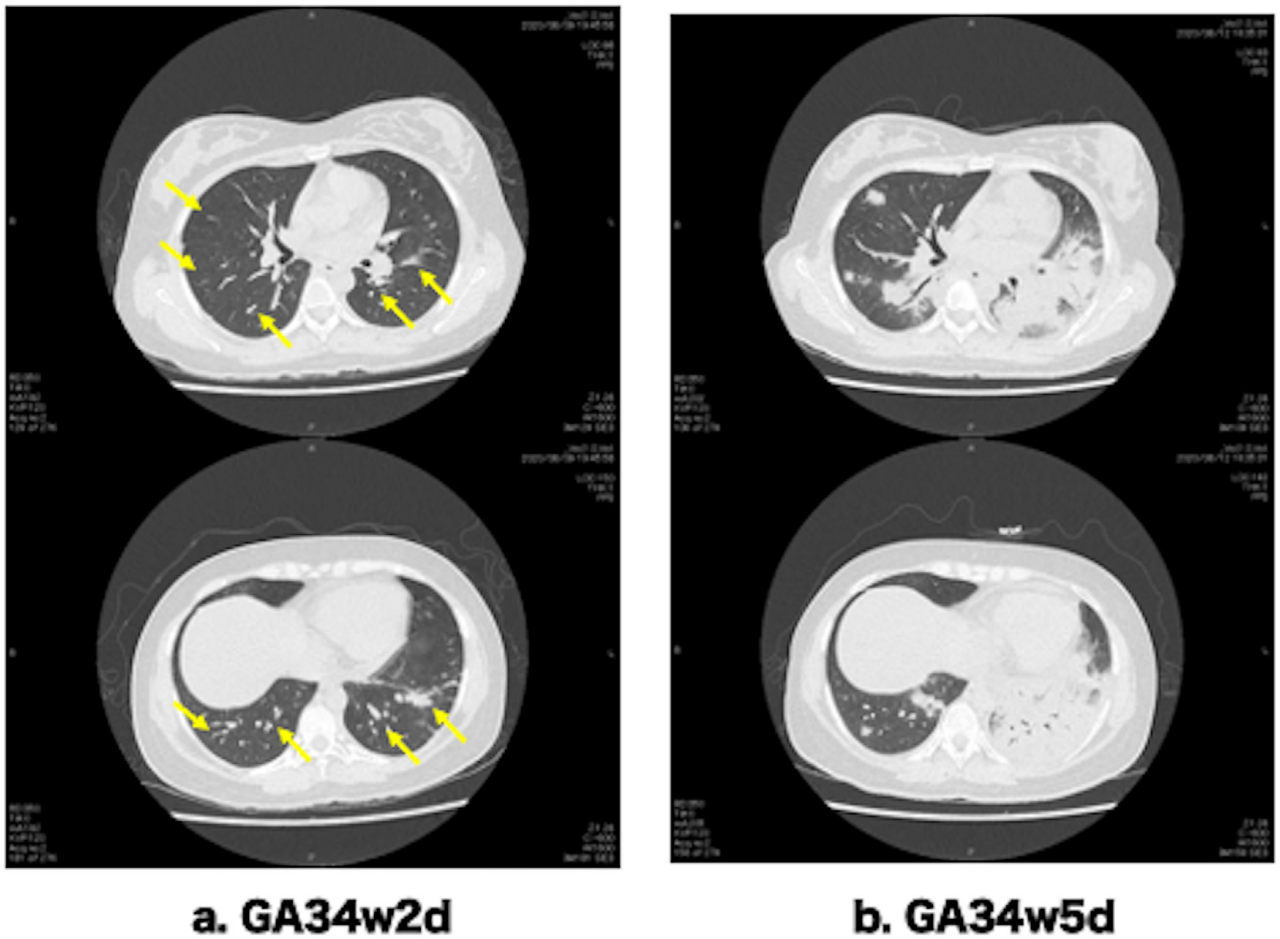
(a) On admission of the patient at 34 weeks and 2 days of gestation, a chest computed tomography scan revealed bilateral multifocal ground-glass opacities with partial consolidation (arrows). (b) Chest computed tomography at 34 weeks and 5 days of gestation showed rapid deterioration.

**Fig. 2:**
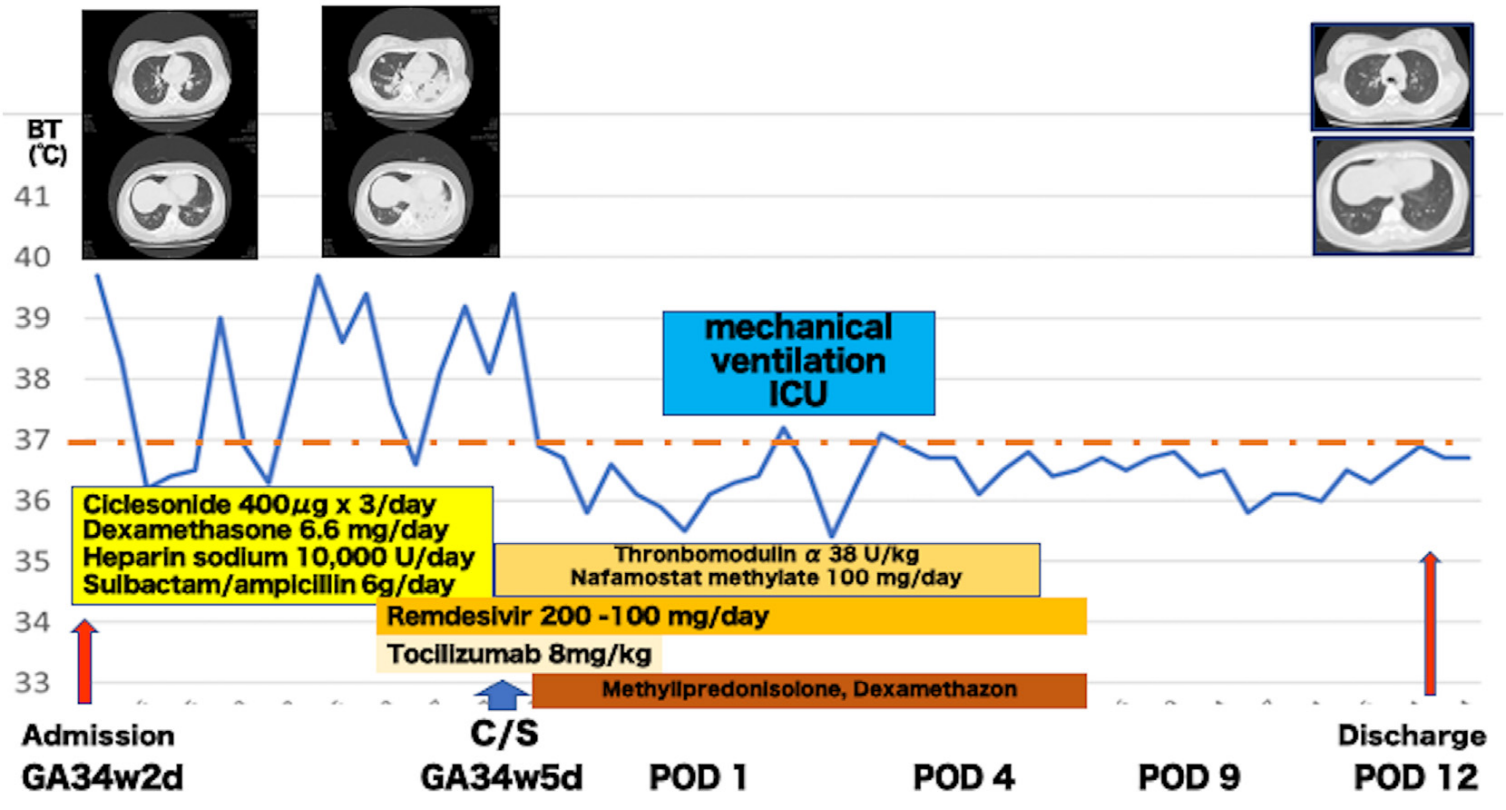
Timeline of the patient's treatment.

A 29-year-old primiparous woman (body mass index 27.5 kg/m^2^) was referred to university hospital at 34 weeks and 2 days of gestation because of a positive throat swab for severe acute respiratory syndrome coronavirus 2 on quantitative real-time polymerase chain reaction. The patient had a 4-day history of fever and dyspnea. On admission, her body temperature was 39.0 °C and saturation O_2_ (SpO_2_) was 95% (room air). A chest computed tomography (CT) scan revealed bilateral multifocal ground-glass opacities with partial consolidation, corresponding to COVID-19 pneumonia (Fig. 1). Laboratory data showed a white blood cell (WBC) count of 8800/μL, lymphocyte 12.4%, C-reactive protein (CRP) 3.54 mg/L, D-dimer 2.3 μg/mL (<1.0 μg/mL), and IL-6487 pg/mL (<4.0). Maternal laboratory values during the course of treatment are presented in Table 1, and the timeline of her treatment is detailed in Fig. 2. Ciclesonide inhalation 400 μg/day, dexamethasone 6.6 g/day, heparin sodium 10,000 U/day, and sulbactam/ampicillin 6 g/day were initiated. On the second day after admission, the patient continued to have high fever with chill and shivers, and her dyspnea worsened. SpO_2_ was 95% with a 1 L/min oxygen flow through nasal cannula. Remdesivir 200 mg/day and tocilizumab 8 mg/kg were administered on the next day. On the fourth day after admission (at 34 weeks and 5 days of gestation), her respiratory condition rapidly worsened, and the SpO_2_ was 94%–95% with a 3–4 L/min oxygen mask. Laboratory data showed WBC count of 9000/μl, lymphocyte 11.5%, CRP 10.41 mg/L, and D-dimer 1.5 μg/mL, and chest CT findings showed rapid deterioration (Fig. 1). The patient's respiratory failure required ventilator or extracorporeal membrane oxygenation (ECMO) management.

**Table 1:** Maternal laboratory values during the course of treatment.

Variable	Normal reference range	Admission	Day 4 (Emergency C/S)	Day 5 (Day after C/S)	Day 9 (5 days after C/S)
WBC count × 10^3^/μL	3.1–8.4	8.8	11.4	12.4	9.5
Neutrophil %	40–69	80.7	85.8	72.0	59.0
Lymphocyte %	26–46	12.4	11.5	7.0	30.0
Hemoglobin mg/dL	11.4–14.6	10.2	9.0	11.0	12.2
Platelet count × 10^3^/μL	138–309	190	172	179	398
Ferritin ng/mL	10–80	41.6		81.5	
CRP mg/L	<0.1	3.54	10.41	11.76	<0.1
Procalcitonin μg/L	<0.05	0.163	0.480	0.645	<0.05
Glucose mg/dL	70–110	56	78	108	94
AST IU/L	7–38	23	29	29	94
ALT IU/L	4–44	16	17	17	43
ALP IU/L	80–260	450	372	376	339
γ-GTP IU/L	32	49	55	59	204
T. Bilirubin mg/dL	0.3–1.2	0.6	0.4	0.3	0.4
LDH IU/L	120–240	202	279	353	346
Albumin g/dL	≥4.0	2.5	2.2	1.7	2.2
eGFR mL/min/1.73m^2^	≥90	111.5	93.7	119.0	130.7
Creatinine mg/dL	≤1.0	0.64	0.61	0.49	0.45
Urea mg/dL	7–23	9	7	10	21
Na mEq/L	137–147	133	137	142	135
KmEq/L	3.5–5.0	3.6	3.0	4.4	4.6
Cl mEq/L	98–108	102	107	113	100
PT sec	10.0–13.5	11.9	11.7	11.0	11.9
aPTT sec	50–100	32	35.6	33.6	34.3
D-Dimer μg/mL	<1.9	1.6	1.5	3.3	2.2
Fibrinogen mg/dL	150–400	532	472		296

C/S; Cesarean section.

It was believed that delivery might decrease maternal oxygen consumption and improve lung mechanics. In addition, it would be easier to manage the patient's breathing after delivery. An emergency cesarean section was performed in the negative-pressure operating room under spinal anesthesia, and a 2565-g male infant was delivered with an Apgar score of 8/8 and pH 7.295 of the umbilical artery. The newborn was admitted to the negative-pressure room of the neonatal ICU. The patient's fever subsided, but her SpO_2_ remained at 94%–95%, even with a 10 L/min oxygen mask on the day after the cesarean section. The patient's respiratory condition deteriorated, and pneumonia findings on chest CT had worsened, showing rapidly increased consolidation with air bronchogram in both lung fields. The patient was subsequently intubated and put on mechanical ventilation at the ICU. The initial conditions for the mechanical ventilation were as follows: tidal volume 400 mL, respiratory rate 16 breaths/min, positive end-expiratory pressure 15 cm H_2_O, and fraction of inspired oxygen (FiO_2_) 100%. The ventilator setting and arterial blood gas analysis are shown in Table 2. The patient's respiratory condition quickly improved; she was successfully taken off mechanical ventilation 4 days after her first day of intubation and was discharged 12 days after cesarean delivery. She did not develop sepsis, renal failure, stroke, thromboembolism or cardiomyopathy during the course of treatment. There was no evidence of maternal or fetal vascular malperfusion or acute or chronic inflammatory pathology in the placenta. The infant's breathing and circulation were stable, and chest X-ray showed no abnormal findings. Nasal swabs for SARS-CoV-2 were negative at 24 and 60 h postpartum.

**Table 2:** Ventilator setting and arterial blood gas analysis.

	Day5 (1 day after C/S)	Day 6 (2 days after C/S)	Day 7 (3 days after C/S)	Day 8 (4 days after C/S)
Ventilator Mode	ASV/PSV–ASV/SIMV	–	–	–
FiO_2_	0.7–0.5–0.45	0.3	0.25	0.25
PEEP cm H_2_O	15	15–10	10	7–5
pH	7.334	7.354		7.423
Pco_2_ mmHg	42.8	41.8		41.5
Po_2_ mmHg	77.0	101.0		93.1
HCO_3_ mEq/L	21.9	22.6		26.6
BE mEq/L	−3.0	−2.2		2.5
SAT %	95.2	97.3		

C/S; cesarean section, ASV; Adaptive Support Ventilation, PSV; pressure support ventilation, SIMV; synchronized intermittent mandatory ventilation.

## 3 Discussion

Our case provides additional evidence that raises concerns regarding the possibility of unfavorable maternal consequences of COVID-19 infection during pregnancy [[5], [6], [7]]. Although most mothers infected with COVID-19 have mild disease that does not require treatment, it has been reported that a number of mothers have been admitted to the ICU and some patients have required ECMO [3,[8], [9], [10]].

On admission, our patient seemed to have mild to moderate disease; however, 3 days later, her disease progressed rapidly, and the respiratory failure was considered to require ventilator or ECMO management. The rationale for early delivery, at 34 weeks of gestation, by cesarean section included the opportunity to safely transport the patient to the operating room and the assumption that delivery could decrease maternal oxygen consumption and improve lung mechanics. In addition, delivery made it easier to manage the patient's breathing. It has been speculated that changes in cardiovascular function and immune response during pregnancy exacerbate COVID-19 infection [11]. Physiological changes during pregnancy reduce the mother's resistance to infection [12]. In addition, anatomical changes (e.g., an increase in the transverse diameter of the thorax with diaphragmatic elevation) lead to maternal vulnerability to hypoxic conditions [13]. Vasodilatation and changes in pulmonary volume can induce mucosal edema and increased upper-airway secretions. Furthermore, changes in cell-mediated immune capacity during pregnancy increase pregnant women's susceptibility to viral infections [14]. These findings have important implications for both the severity and quick progression of the disease and rapid improvement after delivery.

After cesarean section, the patient's fever quickly remitted; however, her respiratory condition deteriorated, and the pneumonia findings on chest CT worsened. Re-expansion of the lungs and increased blood volume in the reflux circulation after delivery might have contributed to the deterioration of these conditions. Accordingly, the patient was placed on mechanical ventilation in the ICU. However, the patient was no longer pregnant and she experienced successful and rapid improvement 4 days after the start of intubation. She was discharged with her baby 12 days after cesarean delivery. The infant had an uneventful course without transplacental or vertical transmission of COVID-19 [15].

The timing of delivery should be determined by considering the progression of acute respiratory distress syndrome and age of gestation. Because the risk factors for obstetric patients of progression to severe disease, ICU care or maternal death remain unknown [3,10], it is unclear whether it is better to wait for maternal decompensation. Our patient was already at 34 weeks of gestation, which was adequate for fetal lung maturation. Delivery before maternal decompensation might have been an option. Pregnant women should be considered vulnerable persons for whom exposure to COVID-19 must be prevented by all means.

This case report provides additional evidence that raises concerns about the unfavorable maternal consequences of COVID-19 infection during pregnancy.

## References

[1] Chen L., Li Q., Zheng D. (2020 Jun 18). Clinical characteristics of pregnant women with Covid-19 in Wuhan, China. N. Engl. J. Med..

[2] Ellington S., Strid P., Van Tong T. (2020 June 26). Characteristics of women of reproductive age with laboratory-confirmed SARS-CoV-2 infection by pregnancy status — United States, January 22–June 7, 2020 weekly. MMWR Morb. Mortal. Wkly. Rep..

[3] Hantoushzadeh S., Shamshirsaz A.A., Aleyasin A. (2020 Jul). Maternal death due to COVID-19. Am. J. Obstet. Gynecol..

[4] Allotey J., Stallings E., Bonet M., for PregCOV-19 Living Systematic Review Consotium (2020 Sep 1). Clinical manifestations, risk factors, and maternal and perinatal outcomes of coronavirus disease 2019 in pregnancy: living systematic review and meta-analysis. BMJ.

[5] Schwartz D.A., Graham A.L. (2020 Feb 10). Potential maternal and infant outcomes from (Wuhan) Coronavirus 2019-nCoV infecting pregnant women: lessons from SARS, MERS, and other human coronavirus infections. Viruses.

[6] Chen H., Guo J., Wang C. (2020). Clinical characteristics and intrauterine vertical transmission potential of COVID-19 infection in nine pregnant women: a retrospective review of medical records. Lancet.

[7] Breslin N., Baptiste C., Miller R. (2020 May). COVID-19 in pregnancy: early lessons. Am. J. Obstet. Gynecol. MFM.

[8] Liu Y., Chen H., Tang K., Guo Y. (2020). Clinical manifestations and outcome of SARSCoV-2 infection during pregnancy. J. Inf. Secur..

[9] Liu Y., Chen H., Tang K., Guo Y. (2020). Clinical manifestations and outcome of SARS- CoV-2 infection during pregnancy. J. Inf. Secur..

[10] Andrikopoulou M., Madden N., Wen T. (2020 Aug). Symptoms and critical illness among obstetric patients with coronavirus disease 2019 (COVID-19) infection. Obstet. Gynecol..

[11] Zaigham M., Andersson O. (2020 Jul). Maternal and perinatal outcomes with COVID-19: A systematic review of 108 pregnancies. Acta Obstet. Gynecol. Scand..

[12] Goodnight W.H., Soper D.E. (2005). Pneumonia in pregnancy. Crit. Care Med..

[13] O’Day M.P. (1997). Cardio-respiratory physiological adaptation of pregnancy. Semin. Perinatol..

[14] Nelson-Piercy C. (2015). Respiratory disease. Handbook of Obstetric Medicine.

[15] Vivanti A.J., Vauloup-Fellous C., Prevot S. (2020 Jul 14). Transplacental transmission of SARS-CoV-2 infection. Nat. Commun..

